# Two novel mutations in the *DNAH11* gene in primary ciliary dyskinesia (CILD7) with considerable variety in the clinical and beating cilia phenotype

**DOI:** 10.1186/s12881-020-01171-2

**Published:** 2020-11-26

**Authors:** Rüdiger Schultz, Varpu Elenius, Heikki Lukkarinen, Tanja Saarela

**Affiliations:** 1 Tampere University Hospital, Allergy Centre, PB 2000, 33521 Tampere, Finland; 2 Department of Pediatrics, Turku University Hospital, Kiinanmyllynkatu 4-8, 20520 Turku, Finland; 3 Department of Clinical Genetics, Kuopio University Hospital, PB 1, 70029 Kuopio, Finland

**Keywords:** PCD, *DNAH11* gene, Compound heterozygosity, PICADAR, Combined diagnostics, Mild disease

## Abstract

**Background:**

Diagnosis of primary ciliary dyskinesia (PCD) still remains a challenge, especially with mutations in the Dynein Arm Heavy Chain 11 (*DNAH11*) gene. Classical diagnostic measures like Transmission Electron Microscopy (TEM) are not applicable for mutations in the *DNAH11* gene since ultrastructural defects of the ciliary apparatus are absent. Novel mutations encoding for PCD appear all the time with considerable variation in the clinical picture, making it necessary to update data bases and guidelines for PCD diagnostics.

**Methods:**

In this study we examined two unrelated, Finnish families with symptoms of PCD applying the clinical scoring system: Primary ciliary dyskinesia Rule (PICADAR), high speed video microscopy analysis (HSVMA) for ciliary movement, a commercially available gene panel analysis and nasal Nitric Oxide (nNO) measurements if applicable.

**Results:**

Two, likely pathogenic variants in the *DNAH11* gene (c.2341G > A, p. (Glu781Lys) ja c.7645 + 5G > A) were detected. In the first family, compound heterozygous mutations led to disease manifestation in two of 4 children, which showed a similar phenotype of cilia beating pattern but marked differences in disease severity. In the second family, all three children were homozygotes for the c.2341G > A p.(Glu781Lys) mutation and showed a similar degree of disease severity. However, the phenotype of cilia beating pattern was different ranging from stiff, static cilia to a hyperkinetic movement in one of these children.

**Conclusions:**

In this study we describe two Finnish families with PCD, revealing two novel mutations in the *DNAH11* gene which show considerable variety in the clinical and beating cilia phenotype. The results of this study show the clinician that PCD can be much milder than generally expected and diagnosis demands a combination of measures which are only successful in experienced hands. Chronic and repeatedly treated wet cough should raise suspicion of PCD, referring the patient for further diagnostics to a specialised PCD centre.

**Supplementary Information:**

The online version contains supplementary material available at 10.1186/s12881-020-01171-2.

## Background

Primary ciliary dyskinesia (PCD) is an inherited, genetically and clinically heterogeneous disorder which causes disturbances in the movement of beating cilia. In the sinobronchial epithelium PCD leads to impaired mucociliary clearance. Affected patients suffer from chronic upper and lower respiratory tract infections which may lead in later life to the development of bronchiectasis and serious damage to the lungs [1]. Prevalence of PCD is estimated to be in the range of 1:4000 to 50.000. This gap of disease prevalence probably reflects rather the impaired availability of proper diagnostics than variability of genetics among different ethnic groups [2]. However, some degree of variability can be explained by a higher prevalence in societies with a high degree of consanguinity [3].

Over 300 genes are involved in the function and morphology of the ciliary apparatus, from which at least 40 mutations of genes are known to be disease-causing [4]. However, currently one third of patients with PCD have no mutations in these genes [5]. The *DNAH11* gene encodes for an axonemal outer dynein arm heavy chain that is expressed primarily in the lungs [6]. Mutations in this gene are associated with PCD but normal cilia ultrastructure [7, 8]. Like most known ciliary disorders, mutations in the *DNAH11* gene are inherited in an autosomal recessive trait.

The clinical phenotypes of *DNAH11* mutations are similar to other mutations causing PCD, including mild, transient tachypnoea, persistent rhinitis or a blocked nose in the neonate period leading often to feeding difficulties. During childhood, the development of chronic middle ear infections and rhinosinusitis is the prominent feature of the disorder, accompanied by a typical chronic, wet cough due to persistent, bacterial bronchitis. In adulthood, the same symptoms persist but increasingly consolidations, atelectasis and bronchiectasis appear in HRCT investigations and bronchial colonisation of *Pseudomonas aeruginosa* may be present [9]. Patients of both genders may also suffer from sub- or infertility [7]. Only 40–50% of patients present with *situs inversus* or heterotaxy [10].

Patients with PCD are still underdiagnosed or diagnosed too late [2]. The clinician therefore should also be aware of atypical phenotypes. Tools like the PICADAR questionnaire may help to raise the level of suspicion. In the past, electron microscopy has been the gold standard for the diagnosis of PCD. However, patients who carry *DNHA11* gene mutations show the typical clinical picture of PCD but morphological defects in the ultrastructure of cilia are absent which demands a different diagnostic approach. Today a growing number of centres use a combined, complementary approach, including measurement of nNO and HSVMA as first line diagnostics [2, 11].

In this study we describe five patients in two unrelated-, Finnish families from the same area of Western-Finland, which were investigated for PCD on suspicion of one member of each family employing the PICADAR questionnaire after Behan et al. [12], nNO measurements, HSVMA and genetic testing.

## Methods

We examined five children suspicious for PCD from two families who originate both from the same region in Western- Finland. Table 1 gives a summary of clinical and diagnostic findings of our five index patients.

**Table 1:** Patient Characteristics

No	Gender/Age/Family	nNO	HSVMA	Genetics (Mutation)	Clinical Picture	PICADAR
1	Female/ 10 years **Family 1**	73.9–98 ppb	Almost static, minimal, residual movement or vibration of cilia tips.	*DNAH11*: HET c.2341G > A p. (Glu781Lys) and HET c.7645 + 5G > A	perinatal onset of rhinorrhea, recurrent otitis media, wet cough, blocked nose, rhinosinusitis, asthma bronchiale, *situs inversus*	10/14
2	Male/ 16 years **Family 1**	301 ppb	Slow, stiff and uncoordinated movement, low bending capacity	*DNAH11*: HET c.2341G > A p. (Glu781Lys) and HET c.7645 + 5G > A	school-age onset with recurrent rhinosinusitis and chronic wet cough	3/14
3	Female/ 6 years **Family 2**	45.8 ppb	Minimal, residual ciliary movement	*DNAH11*: HOM c.2341G > A *p*. (Glu781Lys)	early onset and persistent wet cough, perineal rhinitis, recurrent otitis media, obstructive bronchitis, atopic dermatitis, egg allergy, allergic asthma bronchiale	4/14
4	Female/ 4 years **Family 2**	–	Hyperkinetic, ineffective movement	*DNAH11*: HOM c.2341G > A p. (Glu781Lys)	early onset recurrent otitis media, wet cough, *situs inversus*	10/14
5	Female/ 3 years **Family 2**	–	Minimal, residual ciliary movement	*DNAH11*: HOM c.2341G > A p. (Glu781Lys)	neonatal onset of rhinorrhea, slimy and persistent wet cough and recurrent otitis media	6/14

### PICADAR questionnaire

Patients were interviewed and scored for probable PCD, using the PICADAR questionnaire (see additional file 1).

### Nasal nitric oxide analysis (nNO)

For nNO a CLD 88sp analyser equipped with a Denox 88 module for flow control was used (Eco Physics, Dürnten, Switzerland). If cooperativity was established, three consecutive trials were taken, from which the highest value was recorded. Nasal nitric oxide analysis was repeated on two different occasions.

### Collection of respiratory epithelial cells

After a two week course of oral amoxicillin-clavulanic acid, ciliary cells were obtained by brushing the inferior turbinate using an interdental brush of 0.6 mm size (Topdental Products Ltd., England, UK). Several strips of epithelial, ciliated cells were dropped into an Eppendorf tube containing the cell nourishing medium DMEM (Dulbecco’s Modified Eagle Medium, Thermo Fisher Scientific Oy, Espoo, Finland). The specimen was transported in a cooled container to be analysed two hours after brushing. Before investigating the beating cilia, the medium was warmed up to 37 °C to mimic an optimal, in vivo-like environment during analysis.

### High speed video microscopy analysis (HSVMA)

The warmed up samples were evaluated under a differential-interference microscope (Zeiss, Oberkochen, Germany) at × 1000 magnification and cilia beat was recorded with a digital high-speed video (DHSV) camera (Hamamatsu Orca Flash 4.0 Hamamatsu, Japan) with a frame rate of 400 Hz. DHSV video sequences were played back frame by frame and cilia beat frequency (CBF) was determined by calculating the mean of all recorded cilia beat cycles and the cilia beating pattern (CBP) was determined by two independent expert operators. DHSV was repeated on two different occasions.

### Genetic testing

The venous blood samples of the Tampere University Hospital index patients were taken after written informed consent was obtained. The samples were sent to an accredited laboratory (Blueprint Genetics, Espoo, Finland) for gene panel analysis. Using NGS methods, the coding regions, splice junctions and selected non-coding, deep intronic variants of 36 genes associated with primary ciliary dyskinesia were analysed. The panel (The Blueprint Genetics PCD panel version 3, 2018) included sequence analysis and copy number variation analysis of the following genes: *ARMC4, C210RF59, CCDC103, CCDC114, CCDC39, CCDC40, CCDC65, CCNO, CENPF, CFTR, DNAAF1, DNAAF2, DNAAF3, DNAAF5, DNAH1, DNAH11, DNAH5, DNAI1, DNAI2, DNAL1, DRC1, DYX1C1, GAS8, HYDIN, INVS, LRRC6, NME8, OFD1, PIH1D3, RPGR, RSPH1, RSPH3, RSPH4A, RSPH9, SPAG1* and *ZMYND10.*

For interpretation of sequence variants, the laboratory followed the Blueprint Genetics Variant Classification Schemes modified from the guidelines of the American College of Medical Genetics and Genomics 2015 [13]. Both families were referred to genetic counselling which was carried out at the Tampere University Hospital, Department of Clinical Genetics, by a clinical geneticist. Thereafter, the parents and asymptomatic siblings were tested for familial variants-, in the same laboratory.

### Exclusion of cystic fibrosis

We excluded CF by demonstration of normal sweat tests. In addition, the PCD mutation panel (Blueprint genetics, Medical Lab, Espoo, Finland) also covered frequent CFTR mutations (85% of all CF cases), making the existence of CF as a comorbidity very unlikely.

## Results

Patient 1 and 3 were examined for PCD due to a long history of wet cough and chronic otitis media. After being diagnosed positive for PCD, all other family members were investigated too, undergoing anamnestic interviewing, cilia brushings and HSVMA as well as genetic testing and nNO if applicable. Two of our patients were diagnosed with *situs inversus* (patient 1 and 4) but none of the patients developed bronchiectasis resulting partly in a particularly low score on the PICADAR questionnaire (see additional file 1). Also nasal polyps could not be detected. Patient 1 and 3 were diagnosed for asthma bronchiale. All patients had a history of chronic wet cough but none of the patients suffered from episodes of pneumonia. Cystic fibrosis (CF) could be excluded by sweat tests, additionally, the PCD mutation panel (Blueprint genetics, Espoo, Finland) also covered frequent CFTR mutations (85% of all CF cases), making the existence of CF as a comorbidity very unlikely. In family 1 the clinical picture of the patients differed considerably. While patient 2 had a much milder pathogeny and late onset disease with mainly recurrent episodes of rhinosinusitis and wet cough, his younger sister (patient 1) demonstrated typical early onset symptoms of PCD with chronic otitis, rhinosinusitis and chronic wet cough. Interestingly, in family 1, patient 2 showed normal values of nasal nitric oxide while his sister (patient 1) typically displayed with values under 100 ppb. The beating pattern of cilia in both patients of family 1 was highly abnormal. While patient one had static cilia, patient 2 showed minimal, stiff and uncoordinated movement of cilia. The clinical picture of PCD in the patients of family 2 (patient 3–5) was much more homogeneous than in family 1. All children had a high morbidity and suffered from recurrent otitis media and wet cough. Patient 5 also presented with neonatal rhinorrhea and a blocked nose, leading to feeding difficulties straight after delivery. However, the ciliary beating pattern differed in the range of almost static, minimal movement (patient 3 and 5) to rapid, hyperkinetic strokes (patient 4). In family 2 only the oldest child (patient 3) was able to perform with nNO measurements and revealed typical low values as described with PCD (< 50 ppb).

**Fig. 1:**
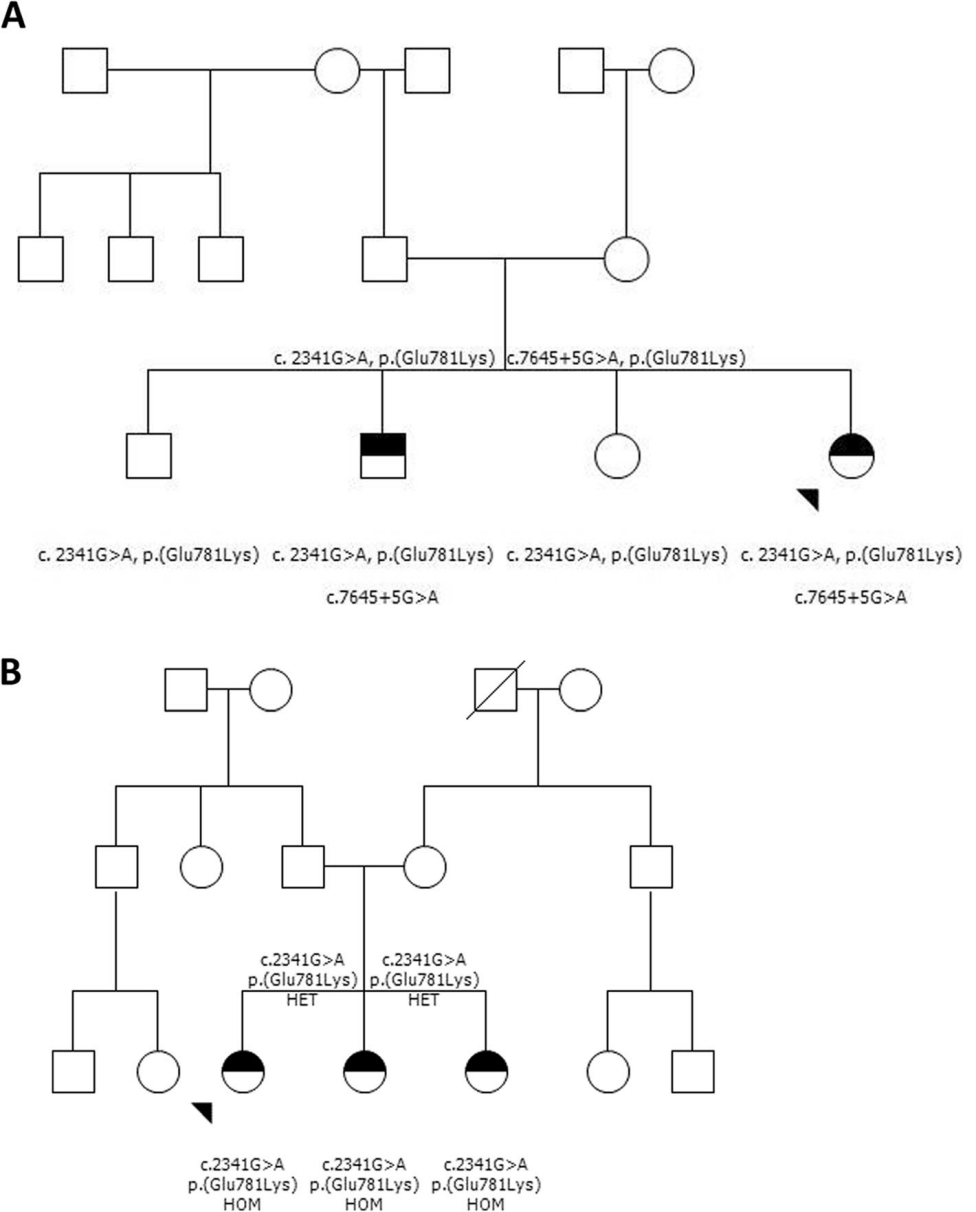
**a** Pedigree of family 1 with one female and one male offspring affected. Two novel mutations were detected in the DNAH11 gene: c.2341G > A p. (Glu781Lys) ja c.7645 + 5G > A, demonstrating compound heterozygosity. **b**: Pedigree of family 2. All three children (female) were affected. The novel mutation c.2341G > A p. Glu781Lys was first detected in family 1. HET = heterozygous DNAH11 gene mutation. HOM = homozygous DNAH11 gene mutation

The genetic testing of patients in family 1 revealed two compound heterozygous variants in the *DNAH11* gene: c.2341G > A, *p. (Glu781Lys) ja* c.7645 + 5G > A (Fig. 1a). All patients in family 2 were homozygous for the *DNAH11* gene variant: c.2341G > A, *p.* (Gly781Lys, Fig. 1b). There are 6 adult individuals heterozygous for the missense variant c.2341G > A, p. (Glu781Lys) in the Genome Aggregation Database (gnomAD). It is predicted deleterious by in silico predictions (Polyphen, SIFT). The splice region variant c.7645 + 5G > A has not been observed in the gnomAD reference population cohorts. It is predicted to weaken the adjacent splice donor site in silico predictions (Alamut Splicing). Neither of the variants have been previously reported in the literature of databases (ClinVar [14], HGMD Professional 2020.3).

## Discussion

Here we describe two novel variants in the *DNAH11* gene causing abnormal movement of beating cilia and clinical disease of PCD in two Finnish families, originating from the same area in Finland. The laboratory classified both variants as likely pathogenic, considering the association between the gene and the patients´ phenotype, rarity in healthy control populations and in silico predicted pathogenicity.

The segregation analysis showed that the two variants occur in trans in the studied individuals (Fig. 1a, b), and they were identified in trans (Fig. 1a) or homozygous (Fig. 1b) in siblings with the same phenotype. The heterozygous carriers of the c.2341G > A, p. (Glu781Lys) variant and the c.7645 + 5G > A variant were healthy and asymptomatic. Thus, the segregation of the variants supported recessive inheritance and pathogenicity of the variants in both families (Fig. 1a and b).

Consanguinity between the two families could not be established. However, all individuals heterozygous for the missense variant c.2341G > A, p. (Glu781Lys) in the Genome Aggregation Database (gnomAD) were in the Finnish control population cohort. Since Finland is considered to be genetically isolated, through the founder effect, the variant c. 2341G > A, p.(Glu781Lys) could be enriched in Finland [15]. Pathogenic variants of *DNAH11* have previously been reported to be truncating (66%) or missense (34%) and have been exclusively seen in patients with a clinical phenotype of PCD, normal ciliary ultrastructure and recessive inheritance [7, 16]. In the first family (Fig. 1a) two of 4 children were affected with compound heterozygosity and these patients showed marked variability in disease severity. The clinical course of patient 1 revealed typical features for PCD with perinatal onset and aggravating symptoms during early childhood, whereas patient 2 had a much milder course of the disease with onset just before school age suffering merely from rhinosinusitis and chronic wet cough. This patient had also a normal nitric oxide count and would probably not have been suspicious for PCD on account of his medical record or PICADAR questionnaire. Because his sister was diagnosed first, he was investigated like all other family members and appeared positive only through HSVMA and genetic testing. In the second family (Fig. 1b) all of their 3 offspring were homozygous for the mutation c.2341 G > A, p. (Glue781Lys) and had all the same severity of disease. However, during HSVMA the beating pattern differed markedly in the range of almost static, minimal movement to rapid, hyperkinetic strokes which is in concert with the observations of Schwabe and Lucas et al. [7, 8]. Only one of the children of family 2 was cooperative enough to perform in nasal nitric oxide measurements, which were characteristically low (< 50 ppb).

Concerning the clinical phenotype of PCD up to 85% of individuals have a history of neonatal onset with unexplained respiratory distress and/or rhinorrhea [2]. This is taken into account in the PICADAR questionnaire (see additional file 1) which helps to raise suspicion for PCD in classical cases. If neonatal distress and *situs inversus* or heterotaxy are absent the score drops rapidly which probably draws the patient out of focus for PCD. Actually, only two of our five patients (patients 1 and 4) received a PICADAR score over 6/14. Considering further diagnostics TEM is not an option with mutations in the DNAH11 gene since this phenotype doesn’t reveal morphological changes of the ciliary apparatus. In addition, an increasing number of PCD variants have been detected (at least 30% of all PCD variants) with normal ultrastructure of cilia [16, 17] questioning the use of TEM in first line diagnostics. Therefore, the use of nasal nitric oxide measurements as the first line diagnostic tool in suspected PCD has been suggested [11]. Especially in children this test demands a high degree of compliance and can be only rarely performed under the age of 6. It has also been of notice that with mutations in the *DNAH11* gene, values of nasal nitric oxide might be in the normal range, as we have shown to be the case with patient 2. This has been reported also by other investigators, especially with the hyperkinetic phenotype of *DNAH11* mutations. In addition, false positive results can also be observed with cystic fibrosis, nasal polyposis and acute upper airway infections [2]. HSVMA is a good choice for first line diagnostics in PCD although it has its own pitfalls and is valuable only in experienced hands. Upper airway infections, other chronic lung diseases like asthma with nasal polyps and cystic fibrosis have an impact on beating cilia. Before the patient is investigated, he or she should be therefore treated with an oral course of antibiotics for two weeks. Most physicians use a broad-spectrum oral antibiotic like amoxicillin plus clavulanic acid or alternatively a cephalosporin to target the common respiratory pathogens. Normally beating cilia rules out PCD. In positive cases a second pathological result should be attained before proceeding to further diagnostics and treatment. Genetic testing has become easily available and prices for the analysis are dropping. At the time of diagnosis, our panel contained 36 mutations but new mutations are appearing constantly in the literature. At the present, approximately one third of diagnosed PCD patients do not have mutations in the listed genes. We therefore suggest that in cases suspicious for PCD and regardless of the score in the PICADAR questionnaire, a combination of diagnostic tools should be used implying HSVMA together with nasal nitric oxide (if possible) and genetic testing.

## Conclusion

This study identified two novel mutations in the *DNAH11* gene which show considerable variety in the clinical picture of PCD and phenotype of beating cilia. Therefore, mutations in the *DNAH11* gene may constitute a considerable diagnostic challenge with low PICADAR scores, normal values of nasal nitric oxide measurements, hyperkinetic movements instead of static cilia and normal ultrastructure. This should remind the clinician that the clinical picture of PCD can be much milder than generally assumed and that for every child with chronic wet cough, a combination of diagnostic tools is needed to rule it out, as recommended in a recent update of guidelines [18].

## Supplementary Information

**Additional file 1.** PICADAR Questionnaire after Behan et al. [12].

## Data Availability

All data gathered, analysed and compiled in this study are included in the manuscript. Patient videos of cilia movement and the Blueprint Genetics laboratory report are available from the corresponding author on reasonable request.

## Abbreviations

CILD7: Ciliary Disorder Number 7
PCD: Primary ciliary dyskinesia
*DNAH11*: Dynein Arm Heavy Chain 11
TEM: Transmission electron microscopy
PICADAR: PrImary CiliAry DyskinesiA Rule
CFTR: Cystic Fibrosis Transmembrane Conductance Regulator
