# Supplementary material for: Two novel mutations in the DNAH11 gene in primary ciliary dyskinesia (CILD7) with considerable variety in the clinical and beating cilia phenotype
Source: BMC Med Genet. 2020 Nov 26;21:237. doi: 10.1186/s12881-020-01171-2 (PMC7690114; doi:10.1186/s12881-020-01171-2)
Supplement: Supplementary file 1 — Additional file 1. PICADAR Questionnaire after Behan et al. [12]. [file 12881_2020_1171_MOESM1_ESM.docx]

**Additional File 1**

**PICADAR Questionnaire after Behan et al. (12)**

| Does the patient have a daily wet cough that started in early childhood? | **Yes** – complete PICADAR  **No** – **STOP** PICADAR is not designed for patients without wet cough | |
| --- | --- | --- |
| 1. Was the patient born pre-term or full term? | Term | 2 |
| 2. Did the patient experience chest symptoms in the neonatal period (e.g. tachypnoea, cough, pneumonia)? | Yes | 2 |
| 3. Was the patient admitted to a neonatal unit? | Yes | 2 |
| 4. Does the patient have a situs abnormality (situs inversus or heterotaxy)? | Yes | 4 |
| 5. Does the patient have a congenital heart defect? | Yes | 2 |
| 6. Does the patient have persistent perennial rhinitis? | Yes | 1 |
| 7. Does the patient experience chronic ear or hearing symptoms (eg. Glue ear, serous otitis media, hearing loss, ear perforation)? | Yes | 1 |

**Total score=**

**Score interpretation**

Patients with a score ≥ 10 have a 90% possibility of testing positive for PCD

Patients with a score ≥ 6 have a 24% possibility of testing positive for PCD
